# Adverse reactions following COVID-19 vaccination: An Ecuadorian experience

**DOI:** 10.1016/j.amsu.2021.103044

**Published:** 2021-11-18

**Authors:** Emanuel Vanegas, Karla Robles-Velasco, María F. Osorio, María José Farfán Bajaña, Zouina Sarfraz, Azza Sarfraz, Juan Carlos Fernández Cadena, Derly Madeleiny Andrade Molina, Matias Panchana Lascano, Ivan Cherrez-Ojeda

**Affiliations:** a Universidad Espíritu Santo, Samborondón, Ecuador; b Respiralab Research Group, Guayaquil, Ecuador; c Pediatrics and Child Health, The Aga Khan University, Karachi, Pakistan; d Research & Publications, Fatima Jinnah Medical University, Lahore, Pakistan; e Omics Sciences Laboratory, Universidad Espíritu Santo, Samborondón, Ecuador

**Keywords:** Adverse reactions, COVID-19, Healthcare workers, Latin America, Vaccination

## Abstract

**Background:**

Limited large-scale studies have been conducted to investigate the adverse effects of COVID-19 vaccine in Latin America, particularly among the healthcare worker (HCW) population in Ecuador. The objective of this study was to assess a cohort of Ecuadorian healthcare workers for adverse reactions following vaccination with the Pfizer-BioNTech vaccine.

**Methods:**

We conducted an observational cross-sectional study to assess the potential adverse reactions to the Pfizer-BioNTech COVID-19 vaccine among a sample of healthcare workers (HCWs) in the city of Guayaquil, Ecuador, from March to May 2021.

**Results:**

The sample comprised 1291 patients, with a mean age of 39.3 years (SD, 13.5). In general, 79% (N = 1020) of participants presented an adverse effect of any type at first dose, while 75.1% (N = 969) did so at the second dose. Pain at the puncture site was the most common adverse effect overall after either the first (68.4%) and second (55.6%) dose. Regarding anaphylaxis, no participant developed the condition after the first dose, and only 0.2% (N = 2) developing it at the second dose. No fatalities were reported.

**Conclusion:**

Our findings suggest that adverse reactions following COVID-19 vaccination with the Pfizer-BioNTech vaccine are relatively common, albeit often mild and self-limited. Consistent with the literature there were few cases of anaphylaxis, and no deaths that could be attributed to the inoculation with the vaccine. We hope our findings can help to reassure the public that benefits of vaccination highly outweigh the risks and contribute to the effort of reducing vaccine hesitancy among those who are concerned about the safety and potential side effects.

## Highlights

- In general, reported adverse reactions were frequent among Ecuadorian healthcare workers, with pain at the puncture site the most common adverse effect.
- Anaphylaxis following vaccination was extremely rare, and we found no fatalities related to the inoculation with either dose of the vaccine.
- We hope our findings can help to reassure the public that benefits of vaccination highly outweigh the risks and contribute to the effort of reducing vaccine hesitancy among those who are concerned about the safety and potential side effects.

## 1 Introduction

The COVID-19 pandemic bears a burden on medical care and economies worldwide, however immunization at the population level provides a way for to reduce future morbidity and mortality [1]. Vaccine hesitancy and reluctance in getting the COVID-19 vaccine and apprehensions about them have been present along the course of vaccination programs and mass immunizations across the world [2]. Factors such as the quick, large-scale production of vaccines, lack of information, and uncertainty about adverse reactions in the public's eye as well about myths spreading through media channels have given rise to suspicion and fear in the Latin American population [2]. Limited large-scale studies have been conducted to investigate the adverse effects of COVID-19 vaccine in Latin America, particularly among the healthcare worker (HCW) population in Ecuador. The objective of this study was to assess a cohort of Ecuadorian healthcare workers for adverse reactions following vaccination with the Pfizer-BioNTech vaccine.

## 2 Methods

We conducted an observational cross-sectional study to assess the potential adverse reactions to the Pfizer-BioNTech COVID-19 vaccine among a sample of healthcare workers (HCWs) in the city of Guayaquil, Ecuador, from March to May 2021. All individuals involved were part of the first phase of the national COVID-19 vaccination plan in our country and were contacted through a local registry established by a local private university. In the first telephone call, potential participants were explained about the purpose of the study, and only after voluntary informed consent was obtained further information was collected. Thereafter, weekly telephone calls were set up to ascertain if adverse reactions had occurred within 14 days of receiving the vaccine.

This study was conducted according to the principles established by the Declaration of Helsinki and was approved by the Expedited Ethics Committee of the Ecuadorian Health Ministry (Approval N° 024–2020). With the information recollected in the survey, personal identification was not possible; as such, anonymity, and personal data protection was guaranteed.

## 3 Results

The sample comprised 1291 patients with a gender distribution of 50.4% female and 41.6% male patients. The mean age of the sample was 39.3 years (SD, 13.5) years (Table 1, Table 2). Around a quarter of the sample had at least one comorbidity (23.3%) and a past medical history compatible with allergic disease (23.1%), where arterial hypertension (11.5%) and drug allergy (10.1%) were reported as the most common conditions, respectively. Furthermore, 28.1% of the studied sample confirmed to be infected with COVID-19 in the past, at least once, no more than a month prior to vaccination. Regarding the vaccine regimen, 73.1% had completed a double dose standard, while 26.9% had only received one dose. The average time between the first and the second dose was 21.5 days (SD, 1.47).

**Table 1:** Demographic and clinical information of surveyed population (n = 1291).

Characteristics	Value % (N)
**Gender**	
Male	41.6 (537)
Female	58.4 (754)
**Comorbidity**	23.3 (301)
Arterial hypertension	11.5 (149)
Diabetes	3.6 (47)
Hypothyroidism	2.2 (29)
Other	6.1 (79)
**History of allergic condition**	23.1 (298)
Drug allergy	10.1 (131)
Allergic rhinitis	8.8 (113)
Food allergy	5.1 (66)
Asthma	3.6 (47)
Atopic dermatitis	1.5 (19)
**Past COVID-19 infection**	28.1 (363)
**Vaccine doses received**	
Single dose	26.9 (347)
Double dose	73.1 (944)
**Outpatient self-medicated**	4.4 (57)
FG-Anti-H1	4.3 (55)
Acetaminophen	0.1 (1)
Antibiotics	0.1 (1)
**Medical attention**	1.5 (19)
FG-Anti-H1	0.5 (7)
Acetaminophen	34.2 (442)
Other NSAIDs	3.5 (45)

**Notes**: FG-Anti-H1, First generation antihistamine H1 receptor; NSAIDs, Nonsteroidal anti-inflammatory drugs.

**Table 2:** Mean age and time between outcomes of interest.

Adverse effect	Mean (SD)
**Age**	39.3 (13.5)
**Days between vaccine doses**	21.5 (1.47)
**Time to present adverse effect after first dose**^a^	6.6 (6.5)
Time for local adverse effect^a^	7.2 (6.6)
Time for systemic adverse effect^a^	6.9 (6.5)
**Time to present adverse effect after second dose**^a^	6.1 (5.4)
Time for local adverse effect^a^	6.5 (5.5)
Time for systemic adverse effect^a^	7.2 (6.2)
**Days presenting adverse effect after onset (first dose)**	2.6 (1.7)
Days for local adverse effect	2.4 (1.6)
Days for systemic adverse effect	2.1 (1.5)
**Days presenting adverse effect after onset (second dose)**	2.4 (1.7)
Days for local adverse effect	2.3 (1.5)
Days for systemic adverse effect	0.5 (0.4)

**Notes**:

a Time is in hours.

In general, 79% (N = 1020) of participants presented an adverse effect of any type at first dose, while 75.1% (N = 969) did so at the second dose (Table 3). Local adverse effects were more common than systemic adverse effects at first (69.4% and 43.1%, respectively) and second (58.3% and 51.6%, respectively) doses. Pain at the puncture site was the most common adverse effect overall after either the first (68.4%) and second (55.6%) dose. In sequence, malaise was the second most common adverse effect overall. Other local adverse effects that were relatively frequent were edema and erythema, whereas headache and fever were notable systemic adverse effects observed after either vaccine dose administration. Regarding anaphylaxis, no participant developed the condition after the first dose, and only 0.2% (N = 2) developing it at the second dose. No fatalities were reported.

**Table 3:** Adverse effects frequencies at first and second doses.

Characteristics	First dose Value % (N)	Second dose Value % (N)
**Adverse effect**	79.0 (1020)	75.1 (969)
**Local adverse effect**	69.4 (896)	58.3 (753)
Pain	68.4 (883)	55.6 (718)
Erythema	5.5 (71)	10.8 (139)
Edema	8.0 (103)	12.8 (165)
Pruritus	0.6 (8)	1.2 (16)
Axillary edema	1.6 (21)	1.1 (14)
**Systemic adverse effect**	43.1 (557)	51.6 (666)
Fever	11.5 (149)	18.0 (233)
Cephalea	18.1 (234)	23.8 (307)
Malaise	19.8 (255)	28.0 (362)
Myalgia	4.6 (60)	6.3 (81)
Arthralgia	4.4 (5)	8.4 (109)
Nausea/Vomiting	2.7 (35)	2.9 (38)
Diarrhea	2.9 (37)	2.3 (30)
Chills	3.5 (45)	6.3 (81)
Fatigue	6.0 (78)	5.4 (70)
Somnolence	2.6 (34)	2.3 (30)
Syncope	0.1 (1)	0.0 (0)
Paresthesia	1.0 (13)	1.2 (15)
Anxiety	0.4 (5)	0.7 (9)
Dizziness	2.7 (35)	2.2 (29)
Epigastric pain	1.3 (17)	0.5 (7)
Generalized rash	0.8 (10)	0.3 (4)
Generalized pruritus	0.6 (8)	06 (8)
Allergic rhinitis	0.2 (3)	0.5 (7)
Petechiae	0.2 (3)	0.3 (4)
Throat itchiness	0.6 (8)	0.2 (2)
Allergic sinusitis	0.4 (5)	0.1 (1)
Facial edema	0.1 (1)	0.0 (0)
Bronchospasm	0.1 (1)	0.0 (0)
Allergic conjunctivitis	0.1 (1)	0.1 (1)
Dermatitis	0.0 (0)	0.1 (1)
Ezcema	0.1 (1)	0.2 (2)
Ocular edema	0.0 (0)	0.1 (1)
Idiopathic urticaria	0.1 (1)	0.0 (0)
Lip swelling	0.1 (1)	0.2 (2)
Tongue swelling	0.1 (1)	0.0 (0)
Lip itchiness	0.0 (0)	0.1 (1)
Facial rash	0.1 (1)	0.0 (0)
Anaphylaxis	0.0 (0)	0.2 (2)

Overall, 79% of the HCWs had an adverse event on the first dose, whereas 75.1% did with the second dose. The local adverse events were more common with the first dose (69.4%) whereas systemic adverse effects were more common with the second dose. As with the CDC report, pain was the most common overall adverse effect. Anaphylaxis was observed in only 2 HCWs post the second dose with no deaths reported.

## 4 Discussion

The centers for disease control and prevention (CDC) finds that among an intervention (N = 2291) and placebo group (N = 2298), 84.7% vaccine recipients reported at least one local injection site reaction [3]. The most frequent local reaction was pain at the injection site. As compared to pain, swelling and redness following either dose of the Pfizer-BioNTech vaccine were less frequently reported [3]. Consistent with previous reports, we found that local adverse reactions were more common than systemic adverse reactions, with pain at the puncture site the most commonly reported event at both doses. It is worth noting that most of the observed reactions were mild or moderate in severity, which coincides with the reports on vaccine safety [1].

On the other hand, serious adverse events are defined as any untoward medical occurrence that results in death, is life threatening, and that requires hospitalization, possibly leading to persistent disability. The percentage of serious adverse events in the vaccine (0.6%) and placebo group (0.5%) have been somewhat similar, with two events namely shoulder injury and lymphadenopathy documented by the FDA post vaccination [3]. Overall, the FDA and CDC reports state that the risk of serious adverse events that involve organ classes are balanced between placebo and vaccine groups. Our study results are consistent with reported literature, as we found no cases of serious adverse events related to the vaccination with either dose.

When immunizing the general population with the Pfizer BioNTech vaccine, anaphylaxis, which is a severe, rapid-onset, multisystem allergic reaction, was reported in 11.1 cases per 1 million infections [4]. This trend was estimated to be up to ten-fold higher than previous, commonly used vaccines. When more estimates were generated, and millions of doses of the COVID-19 vaccines were administered, the updated anaphylaxis rate was determined to be 4.7 cases per 1 million doses. Notably, 90% of the anaphylactic reactions were reported among females, and 81% of the individuals had a history of allergies [4]. Consistent with previous findings, no participants in our study reported an anaphylactic event at the first dose, and only 0.2% developed it at the second dose. Coincidentally, the two patients that developed anaphylaxis were females. In this regard, further studies are needed to accurately assess the allergenic components responsible for anaphylaxis so high-risk patients can be identified and counseled properly to prevent this type of event.

There are increasing concerns among physicians and patients about adverse reactions to the COVID-19 vaccines [5]. Given that the vaccine production is concentrated in the Global North, governments in the Global South do not have their preference in picking vaccines for the citizens [6]. Vaccine hesitancy in Latin America is non-uniform, largely dependent on which vaccine is offered. Data from Latin America suggests that citizens may have imprecise beliefs about public health issues, and the misinformation can lead to hesitancy in acquiring the vaccine [6]. With few studies conducted in Latin America in this area, we hope our findings can help to reassure the public that benefits of vaccination highly outweigh the risks and contribute to the effort of reducing vaccine hesitancy among those who are concerned about the safety and potential side effects.

In light of our findings, there are several limitations worth mentioning. Our sample consisted of healthcare workers who had been informed about the study's purpose before participating, which may have affected their perception of adverse effects. Also, participants were asked to identify past adverse events, so recall bias might have been present. However, to our knowledge this study is among the first to report the occurrence of adverse reactions related to COVID-19 in an Ecuadorian population.

## 5 Conclusions

Our findings suggest that adverse reactions following COVID-19 vaccination with the Pfizer-BioNTech vaccine are relatively common, albeit often mild and self-limited. Local reactions such as pain in the injection site account for the majority of events, while systemic/severe reactions are rare. Consistent with the literature there were few cases of anaphylaxis, and no deaths that could be attributed to the inoculation with the vaccine. We hope our findings can help to reassure the public that benefits of vaccination highly outweigh the risks and contribute to the effort of reducing vaccine hesitancy among those who are concerned about the safety and potential side effects.

## Sources of funding

This work was funded and supported by Universidad Espiritu Santo, Ecuador. The sponsor had no role in the study design, data recollection or statistical analyses.

## Ethical approval

This study was conducted according to the principles established by the Declaration of Helsinki and was approved by the Expedited Ethics Committee of the Ecuadorian Health Ministry (Approval N° 024–2020). With the information recollected in the survey, personal identification was not possible; as such, anonymity, and personal data protection was guaranteed.

## Consent

Voluntary informed consent was obtained.

## Author contribution

Authors have made substantial contributions to conception and design, acquisition, analysis and interpretation of data, have been involved in drafting the manuscript or revising it critically for important intellectual content, and given final approval of the version to be published. All authors read and approved the final version.

## Trial registry number

1. Name of the registry: ClinicalTrials.gov

2. Unique Identifying number or registration ID: NCT05113472

3. Hyperlink to your specific registration (must be publicly accessible and will be checked): https://clinicaltrials.gov/show/NCT05113472

## Guarantor

Ivan Cherrez-Ojeda, Universidad Espíritu Santo, Km. 2.5 Vía La

Puntilla Samborondón 0901–952, Ecuador, Tel +593 999981769, Email ivancherrez@gmail.com

## Declaration of competing interest

The authors declare no conflicts of interest related to this work.

## Abbreviations

HCW: healthcare worker
